# Cost Effectiveness and Budget Impact of the Boston University Approach to Psychiatric Rehabilitation for Increasing the Social Participation of Individuals With Severe Mental Illnesses

**DOI:** 10.3389/fpsyt.2022.880482

**Published:** 2022-05-26

**Authors:** Sarita A. Sanches, Talitha L. Feenstra, Wilma E. Swildens, Jooske T. van Busschbach, Jaap van Weeghel, Thea D. I. van Asselt

**Affiliations:** ^1^Altrecht Institute for Mental Health Care, Utrecht, Netherlands; ^2^Phrenos Center of Expertise for Severe Mental Illness, Utrecht, Netherlands; ^3^Tilburg University, Tilburg School of Social and Behavioral Sciences, Tranzo Scientific Center for Care and Welfare, Tilburg, Netherlands; ^4^University of Groningen, University Medical Center, Department of Epidemiology, Groningen, Netherlands; ^5^University of Groningen, Groningen Research Institute of Pharmacy, Groningen, Netherlands; ^6^Center for Nutrition, Prevention, and Health Services Research, Institute for Public Health and the Environment (RIVM), Bilthoven, Netherlands; ^7^Inholland University of Applied Sciences, Amsterdam, Netherlands; ^8^University of Groningen, University Medical Center Groningen, University Center of Psychiatry, Rob Giel Research Center, Groningen, Netherlands; ^9^Windesheim University of Applied Sciences, School of Human Movement and Education, Zwolle, Netherlands; ^10^Parnassia Psychiatric Institute, The Hague, Netherlands; ^11^University of Groningen, University Medical Center, Department of Health Sciences, Groningen, Netherlands

**Keywords:** Boston University Approach to Psychiatric Rehabilitation, severe mental illness, cost-effectiveness, budget impact, quality of life, QALY, social participation

## Abstract

**Background:**

The purpose of this study was to investigate the cost-effectiveness and budget impact of the Boston University Approach to Psychiatric Rehabilitation (BPR) compared to an active control condition (ACC) to increase the social participation (in competitive employment, unpaid work, education, and meaningful daily activities) of individuals with severe mental illnesses (SMIs). ACC can be described as treatment as usual but with an active component, namely the explicit assignment of providing support with rehabilitation goals in the area of social participation.

**Method:**

In a randomized clinical trial with 188 individuals with SMIs, BPR (*n* = 98) was compared to ACC (*n* = 90). Costs were assessed with the Treatment Inventory of Costs in Patients with psychiatric disorders (TIC-P). Outcome measures for the cost-effectiveness analysis were incremental cost per Quality Adjusted Life Year (QALY) and incremental cost per proportional change in social participation. Budget Impact was investigated using four implementation scenarios and two costing variants.

**Results:**

Total costs per participant at 12-month follow-up were € 12,886 in BPR and € 12,012 in ACC, a non-significant difference. There were no differences with regard to social participation or QALYs. Therefore, BPR was not cost-effective compared to ACC. Types of expenditure with the highest costs were in order of magnitude: supported and sheltered housing, inpatient care, outpatient care, and organized activities. Estimated budget impact of wide BPR implementation ranged from cost savings to €190 million, depending on assumptions regarding uptake. There were no differences between the two costing variants meaning that from a health insurer perspective, there would be no additional costs if BPR was implemented on a wider scale in mental health care institutions.

**Conclusions:**

This was the first study to investigate BPR cost-effectiveness and budget impact. The results showed that BPR was not cost-effective compared to ACC. When interpreting the results, one must keep in mind that the cost-effectiveness of BPR was investigated in the area of social participation, while BPR was designed to offer support in all rehabilitation areas. Therefore, more studies are needed before definite conclusions can be drawn on the cost-effectiveness of the method as a whole.

## Introduction

Individuals with severe mental illnesses (SMIs) experience serious social participation problems. Frequently reported issues are high unemployment rates (1–3) and difficulties participating in daytime activities such as education, unpaid employment or activities outside the home (4, 5). Participation in these activities is important because it facilitates recovery and is desired by most people with SMIs (6–8). Furthermore, competitive employment increases income and alleviates poverty (9). For instance, Levinson et al. (10) found that individuals with SMIs earned -on average- a third less than median earnings in over 19 countries in the Americas, Europe, the Middle East, Africa, Asia and New Zealand.

The low employment rates of people with SMIs not only have personal implications, but also implications for society at large. In the Netherlands and the UK, people with mental health problems represent the largest group (30–40%) of disability benefits claimants (11, 12). Therefore, increasing the social participation of individuals with SMIs may not only facilitate their recovery and improve life satisfaction, but also increase their productivity and reduce benefit payments, which is beneficial for society. Considering these problems, people with SMIs need structured supports with their social participation and this could be achieved through psychiatric rehabilitation. The aim of psychiatric rehabilitation is to “help persons with psychiatric disabilities increase their ability to function successfully and be satisfied in the environment of their choice with the least amount of ongoing professional intervention (13)”. It differs from standard care approaches with regard to its focus on personal goal attainment and (re)gaining various social and personal roles.

Several rehabilitation approaches have been developed to support individuals with SMIs with rehabilitation goals such as increasing their social participation. Some approaches target one aspect of rehabilitation, such as Individual Placement and Support (IPS) for competitive employment (14). Others, such the Strengths model or the Boston University Approach to Psychiatric Rehabilitation (BPR) (13, 15), focus on diverse rehabilitation areas.

Several trials have confirmed that BPR effectively improves social participation and functioning (16–19), and the approach has been implemented in many Dutch Mental Health Care (MHC) facilities. Nevertheless, dissemination is slow and BPR availability remains limited. An important reason for limited implementation is the fear that BPR will be time-consuming and expensive. Cost insights appear essential to further promote implementation, particularly as many MHC institutions are dealing with ongoing budget cuts.

To date, no studies on the cost-effectiveness of BPR have been conducted. Therefore, a randomized controlled trial (RCT) was conducted to investigate the cost-effectiveness and budget impact of BPR compared to an active control condition (ACC) for individuals with SMIs who have a wish for change regarding social participation. An active control condition was chosen to ensure that both conditions received equal amounts of attention and support.

It was hypothesized that BPR would be cost-effective compared to ACC because: (1) based on earlier studies, larger increases in social participation were expected combined with few additional costs, and (2) the methodology's positive effects on functioning would lower MHC costs.

## Methods

### Design

In brief, from 2014–2017, an RCT comparing BPR to ACC was accompanied by quality of life and resource use measurement. A trial-based economic evaluation was conducted alongside this RCT.

Resources were costed using guideline-based unit prices, and cost-effectiveness over the trial follow-up time of 12 months was calculated. For the Budget impact analysis, four scenarios were developed and total costs calculated over a 4 year time horizon. A 4-year time horizon was chosen based on the Dutch and international guidelines (20, 21).

Randomization occurred through block randomization by an independent researcher, and participants were stratified by center and previous work experience. There was no consumer choice. Detailed descriptions of the study protocol and results on BPR effectiveness compared to ACC are presented elsewhere (22, 23).

### Participants

In total, 188 participants with SMIs were recruited at two organizations offering supported and sheltered housing, at three regional MHC centers offering outpatient care [Functional Assertive Community Teams (F-ACT); van Veldhuizen (24)], a team for patients with enduring eating disorders, and an outpatient team for substance addiction. Participants needed to have severe mental illness (a DSM-V diagnosis, long duration of service contact and functional impairments which substantially interfere with or limit major life activities), be between 18–64 years old, and have a wish for change in social participation. Participants were excluded if hospitalized during enrolment, except when treated for severe eating disorders. Participants were randomized to receive either BPR (98 participants) or ACC (90 participants), and were assessed at baseline, and at 6 and 12 months after enrollment.

### Interventions

#### BPR

BPR is a systematic psychiatric rehabilitation approach designed to help individuals with SMIs achieve and retain rehabilitation goals with regard to housing, education, work and social contacts. The approach consists of four phases: exploring, choosing, getting and keeping rehabilitation goals (13, 25). Each phase consists of several techniques that can be used by BPR practitioners to optimally support their patients. BPR is characterized by its person-centered focus: the patient sets the goal and directs the pace of the rehabilitation process and the practitioner has a facilitating role.

BPR was delivered by 28 BPR-practitioners: mental health professionals from different vocational backgrounds who had all received additional training in BPR from the Dutch training institute for BPR, R92 or internal training from BPR trainers licensed by R92.

#### ACC

ACC can be described as treatment as usual with an active component. The mental health practitioners in the ACC group did not work according to a systematic rehabilitation method, however they did receive the explicit assignment of providing support with rehabilitation goals in the area of social participation. Participants in the ACC condition were also offered at least one session every 2 weeks. ACC was delivered by 55 practitioners who also offered active rehabilitative support with participants' personal goals, but without using a structured rehabilitation methodology. These mental health professionals came from vocational backgrounds comparable to the BPR practitioners but had not received specialized training.

In both conditions, participants were offered at least one session every 2 weeks without a predetermined minimum or maximum number of sessions to be completed. Furthermore, practitioners in both conditions were allowed to involve additional resources such as specialized vocational services that are available in most Dutch municipalities for people with problems regarding work participation, including facilities specialized for unemployed persons with mental health problems and a distance to the labor market.

There were no differences between practitioners in ACC and BPR with regard to educational level but ACC practitioners had significantly more years of experience than BPR practitioners [BPR: M (SD) = 15,66 (9,75); ACC: M (SD) = 20,09 (12,22); *p* = 0.007].

### Measures

#### Costs

Costs were assessed according to the Dutch guidelines for economic evaluation (21). Healthcare resource use was obtained at baseline, 6 months and 12 months, using the Treatment Inventory of Costs in Patients with psychiatric disorders (TIC-P) (26), adapted to match the specific context of the target population. For medication costs, prices from the year 2017 based on price information from the National Health Care Institute were applied (27). For other costs, unit prices were taken from the cost manual by Hakkaart-van Roijen et al. (28) [an appendix to the Dutch guidelines for economic evaluation (21)], which were based on 2014 and indexed to 2017 using the national consumer price index (29). Full details on unit prices per type of costs can be found in Appendix 1. The friction cost method was applied for productivity losses (28). The friction cost method is a way of estimating the costs of production loss from the absence of an employee during the period that is needed to fully replace that employee by another one (the friction period). The underlying assumption is that the previous level of productivity will be resumed by the new employee. Standardized friction periods and hourly rates are available from the Dutch guideline for economic evaluations (21). In case of partial absenteeism, the number of absent hours was multiplied by the hourly wage rate. In case of full absenteeism, the job size in hours was multiplied by the hourly rate, up to a maximum of 85 days (the 2014 friction period). In accordance with the guideline, the same procedure was adopted for unpaid work using corresponding prices from the cost manual (28).

Intervention costs were based on the number of sessions and costs per hour for practitioners. Sometimes participants were referred to external job coaches. These are job coaches that are not part of the patients' team or mental health organization but employees that work with a specialized vocational service outside the mental health organization. These external job coaches are supported by local employment services that are available for people with a distance to the labor market in most Dutch municipalities, and also support unemployed persons with mental health difficulties. Because no detailed data on the exact hours spent with these external job coaches were available, the average duration and price for job coaches reimbursed by the Employee Insurance Agency (UWV) was used (8 × € 92 = € 736) (30). The training that BPR practitioners received (initial training) was already provided before the start of the study and practitioners had been working along the lines of this approach with patients other than those participating in the trial. Therefore, the per patient costs of the initial training are low and uncertain and were not included in the total intervention costs. Travel costs were not measured and not included in the analysis since they were deemed small compared to other cost elements.

#### Effectiveness

Generic health status was measured at baseline, 6 months and 12 months, with the 12-item Short Form Health Survey (SF-12) (31), which was transformed into SF-6D scores using the methodology developed by Brazier and Roberts (32). The SF-6D utility scores were subsequently used to derive Quality Adjusted Life Years (QALYs) at 6 and 12 months (33). Social participation over the past 6 months was assessed using the Occupation and Employment subscale of the Birchwood Social Functioning Scale (SFS_OE) (34). The raw SFS_OE score was dichotomized in no employment (scores 0–6) vs. employment (scores = >7).

### Data Analysis

#### Cost Effectiveness Analysis (CEA)

The CEA was performed from a societal perspective. The study had a 12-month time horizon; costs and health outcomes were not discounted (33). Cost-effectiveness outcomes were expressed as incremental costs per percentage change in the dichotomized SFS_OE subscale. Furthermore, a cost utility analysis was conducted resulting in incremental costs per QALY gained. Missing values were estimated using Multiple Imputation in SPSS-25 and aggregated using Rubin's rule (35). Analyses were repeated on individuals with complete data. All CEAs were conducted in SPSS v25 (36).

#### Budget Impact Analyses (BIA)

The BIA was conducted according to the guidelines of Sullivan et al. (20), from a societal perspective and using unit prices as in the CEA. Trial results were extrapolated to a time horizon of up to 4 years and to the entire Dutch population of people with SMIs. Data of participants with complete data were used. The results of the cost-effectiveness analysis were combined with epidemiological data on the size of the target population, and data on BPR program scale and implementation. The BIA was performed for four scenarios: (1) a maximum implementation scenario assuming participation of all eligible patients with SMIs between 18–64 years; (2) An optimistic trial scenario assuming inclusion criteria and participation rates as observed in the study, but adjusting for drop-out, (3) A conservative trial scenario assuming inclusion criteria, participation rates and dropout rates as observed in the study, 4) A scenario assuming participation of only those who receive outpatient care from F-ACT (24) and are between 18–64 years.

Two costing variants were applied, exploring different perspectives and assumptions regarding costs per participant: In variant one, mean resource use per participant was taken from the trial results, and valued from a societal perspective. In variant two, a third party payer (health insurance) perspective was used, and all resource use was valued using actual reimbursement rules.

In contrast to the CEA, for the BIA, initial training costs were included as one-time up-front spending for the BPR scenarios, to reflect the investments needed for introducing it into current care.

#### Uncertainty Analysis

In a dedicated spreadsheet tool, bootstrapping with 1000 replications was performed to assess uncertainty surrounding the cost-effectiveness and cost-utility ratios, and around the differences in mean costs per participant for the BIA. The results are presented in incremental cost-effectiveness planes (CE-planes), and cost-effectiveness acceptability curves (CEACs), while for the BIA the 95%-interquantile ranges were used to present a range of possible values for the total costs of large scale implementation.

## Results

All 188 participants were included in the economic evaluation. There were no significant baseline differences on any of the sociodemographic variables measured (Table 1).

**Table 1:** Sociodemographic characteristics of study participants at baseline.

**Variable**	**BPR (98)**	**ACC (90)**	**Test statistic (df)**	***p***
Age, years: mean (SD)	39.18 (10.68)	40.67 (12.04)	*t*(186) = 0.90	0.372
Gender, female, *n* (%)	40 (40.8)	39 (43.3)	*χ^2^*(1) = 0.12	0.727
Civil status, *n* (%)				
Single / divorced / widowed Married / in partnership	89 (90.8) 9 (9.2)	85 (94.4) 5 (5.6)	*χ^2^*(1) = 0.90	0.344
Living situation, *n* (%)				
Independent	67 (68.4)	62 (68.9)	*χ^2^*(2) = 0.92	0.630
Dependent	30 (30.6)	28 (31.1)		
Educational level, *n* (%)^1^				
Low^2^ Medium^3^ High^4^	40 (40.8) 40 (40.8) 17 (17.3)	35 (38.9) 39 (43.3) 16 (17.8)	*χ^2^*(3) = 1.04	0.792
Working status, *n* (%)				
Paid employment Unpaid work Education	8 (8.2) 33 (33.7) 3 (3.1)	5 (5.6) 35 (38.9) 5 (5.6)	*χ^2^*(1) = 0.47 *χ^2^*(1) = 0.55 *χ^2^*(1) = 0.72	0.494 0.457 0.397
Main diagnosis^5^, *n* (%)				
Psychotic disorder Bipolar disorder Depressive or anxiety disorder Personality disorder Eating disorder Other	59 (60.2) 2 (2.0) 7 (7.1) 8 (8.2) 8 (8.2) 22 (22.4)	54 (60) 4 (4.4) 6 (6.7) 4 (4.4) 5 (5.6) 22 (24.4)	*χ^2^*(5) = 2.95	0.708
Psychiatric symptoms^6^: mean score (SD)	44.25 (13.18)	45.12 (12.56)	*t*(184) = 0.46	0.647
Duration in MHC (in years): mean (SD)	15.75 (9.93)	15.36 (11.63)	*t*(182) = −0.24	0.808
Social functioning^7^: mean score (SD)	125.26 (20.16)	127.92 (23.62)	*t*(186) = 0.83	0.406

1 * (47) OECD, European Union, UNESCO Institute for Statistics. ISCED 2011 Operational Manual: Guidelines for Classifying National Education Programmes and Related Qualifications. OECD Publishing; 2015*.

2 *ISCED level 0 – 2*;

3 *ISCED level 3–5*;

4 *ISCED level 6 – 8*;

5 *According to DSM-IV criteria*;

6 *Brief Psychiatric Rating Scale (BPRS); scores range from 24–168; higher scores indicate more psychiatric symptoms*;

7 *Birchwood Social Functioning Scale (SFS) total score; scores range from 0–223; higher scores indicate fewer impairments*.

### Costs

The average number of sessions did not significantly differ between conditions (BPR: 16.15 (SD = 11.37); ACC: 13.87 (SD = 12.81); t = −1.30, df = 186, P = 0.197). A detailed overview of costs is provided in Table 2. Total intervention costs after 12 months were €551 in BPR and €492 in ACC. Total costs including intervention costs did not significantly differ between BPR and ACC at 12 months (BPR: €12,886; ACC €12,012; 95% CI: €−3.59–€11.43). Types of expenditure in which most costs were made were in order of magnitude: supported and sheltered housing, inpatient care, outpatient care, and organized activities.

**Table 2:** Differences in participation scores and QALYs (top part of table) and total pooled average costs across assessment points by type of expenditure in euros (price level 2017).

	**Baseline**		**6-months**		**12-months**	
	**BPR**	**ACC**	**BPR**	**ACC**	**BPR**	**ACC**
*Primary outcomes*						
SFS_OE^1^	0.48	0.49	0.60	0.59	0.55	0.62
QALY^2^	n.a.	n.a.	0.34	0.34	0.68	0.68
Total costs^3^	n.a.	n.a.	14084	11802	12886	12012
*Healthcare costs*						
Intervention^4^	n.a.	n.a.	275	246	551	492
Additional resources^5^	n.a.	n.a.	56	110	56	110
Inpatient care	3384	5026	1897	1406	2345	2910
Outpatient care	2752	4764	1964	1945	1416	1219
Supp./sheltered housing	7861	8158	7204	6200	5952	5139
Home care	646	124	467	88	658	126
Prescribed medication	128	322	200	180	187	396
Non-prescribed medication	22	12	9	13	42	16
*Costs outside healthcare*						
Organized activities	1317	1251	1148	1002	1157	1361
Social support and informal home care	82	49	64	42	41	60
Unexpected expenditures	49	34	58	49	97	78
*Indirect costs*						
Productivity loss unpaid work	47	158	98	98	97	78
Productivity loss paid work	145	0	643	422	444	234

1 *Dichotomized SFS score: no employment (scores 0 through 6) vs employment (scores 7 and up)*;

2 *QALY is a cumulative measure made up of utility scores at multiple time points. As there is only one time point available at T0, QALYs are not applicable at this time point*;

3 *total costs are not available at baseline because intervention costs and additional costs such as job coaching had not yet started at T0*.

4 *Including costs for supervision*;

5 *Additional support such as external job coaching*.

### Effectiveness

The results on the effectiveness of BPR compared to ACC have been published elsewhere (23), and showed that participants in BPR as well as ACC significantly improved their social participation during the study period. However, BPR was not more effective than ACC on any of the primary or secondary outcome measures. The difference in participation score at 12 months (SFS) was not significant (BPR: 0.55; ACC: 0.62; 95% CI SFS: −0.20–0.08). The difference in QALYs at 12 months was very small and not significant (BPR: 0.68; ACC: 0.68; 95% CI QALY: −0.04–0.03), indicating that the intervention did not have a lasting effect on the quality of life when compared to ACC in this study.

### Cost-Effectiveness

**Figure 1:**
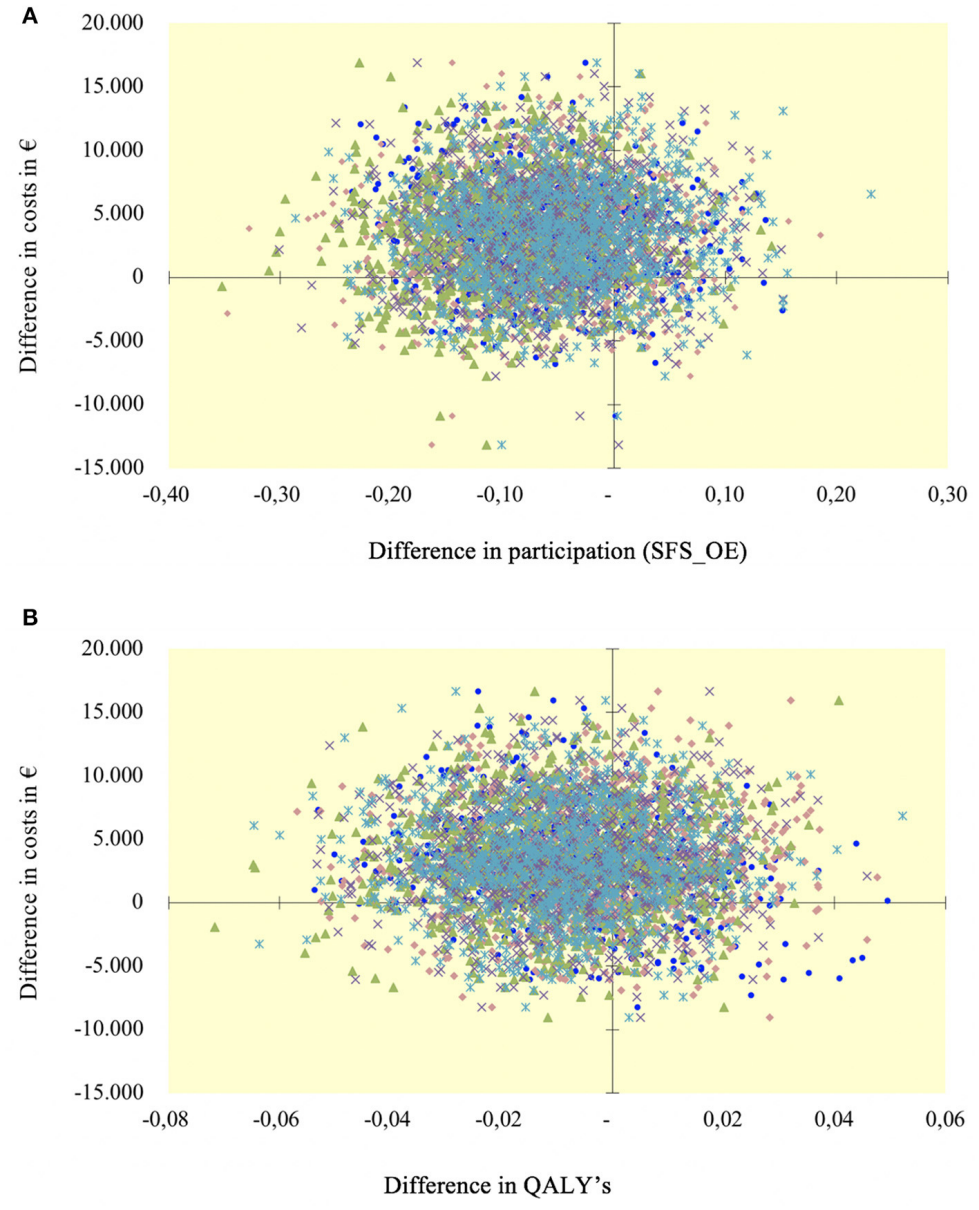
Cost-effectiveness planes (costs are reported in euros). **(A)** Cost per increase in participation at 12 months; **(B)** Cost per increase in QALY at 12 months.

For both effectiveness measures, BPR was not cost-effective compared to ACC at 12-month follow-up. This is highlighted in the incremental CE planes (Figure 1). Because of the minimal difference in QALYs and the negative difference in social participation, it was not informative to calculate incremental cost-effectiveness ratios (ICERs).

**Figure 2:**
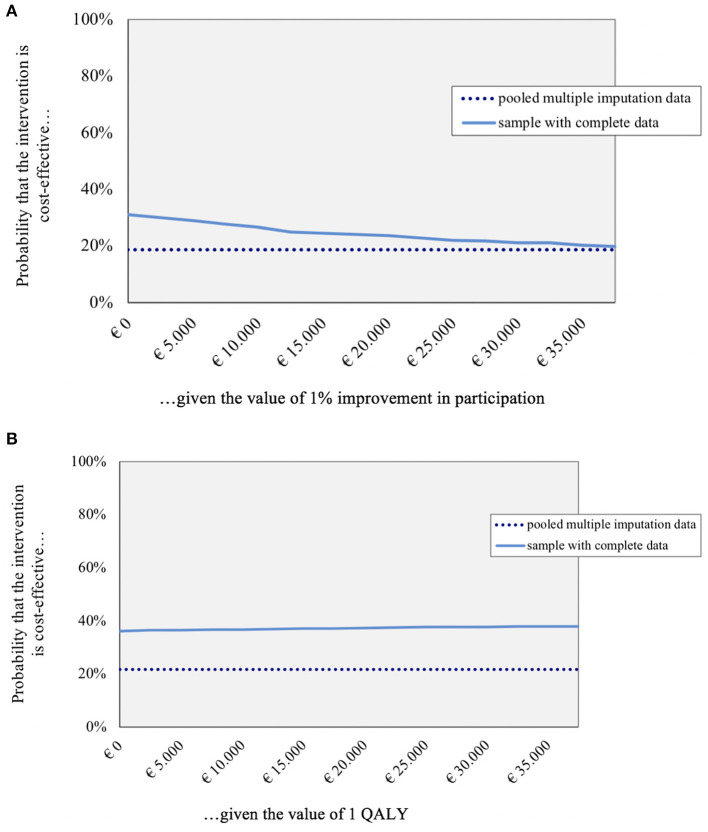
Cost effectiveness acceptability curves for pooled imputed and complete data (costs are reported in euros). **(A)** Bootstrap SFS_OE at 12 months; **(B)** Bootstrap QALY at 12 months.

Figure 2 shows the CEACs. With the SFS as outcome (3a), the probability that BPR is cost-effective varies between 20–31%. With the QALY as outcome, the probability of BPR being cost-effective varies between 36–38%. This underlines the slim chance of BPR being cost-effective compared to ACC in this study sample.

### Complete Data

Complete data on costs and QALYs were available for 77 participants in BPR and 69 in ACC. Complete data on costs and SFS were available for 78 participants in BPR and 70 in ACC. Completers on costs per QALY had significantly fewer psychiatric symptoms than non-completers (*t* = 2.06, df = 54.99, *p* = 0.044). Completers on costs per increase in participation (SFS) were significantly more often male than non-completers χ^2^(1) = 5.00, *p* = 0.025). In both sets of completers there were no significant differences between completers and non-completers concerning duration in MHC, educational level, psychiatric diagnosis, or having paid employment.

Outcomes for completers still indicated that BPR was not cost-effective compared to ACC (dashed lines in Figures 2A,B).

### Budget Impact Analysis (BIA)

Table 3 presents the results for the BIA. The estimated number of participants for the whole of the Netherlands in the various scenarios varied from 2,000 to 16,000 for follow-up years. In the first year it was substantially higher, since it was assumed all persons known with SMIs would be treated during this year, while in the follow-up years only new cases were included for treatment.

**Table 3:** Results for budget impact analysis (price level 2017, in mln euros).

**Perspective**	**scenario**	**Costing**	**Number of individuals (year 1)**	**Year 2 and further**	**Baseline BIA (year 1)**	**Year 2 and further**	**Lower year 2 and further**	**Upper year 2 and further**
Societal	maximum implementation	Trial costs	74100	16200	€ 101	€ 22	–€ 140,	€ 190
Societal	conservative trial based	Trial costs	9386	2052	€ 13	€ 3	–€ 18	€ 24
Societal	Optimistic trial based	Trial costs	56316	12312	€ 77	€ 17	–€ 107	€ 144
Societal	Only F-ACT participants	Trial costs	48906	10692	€ 67	€ 15	–€ 93	€ 125
Healthcare	maximum implementation	Trial costs	74100	16200	€ 51	€ 11	–€ 128	€ 157
Healthcare	conservative trial based	Trial costs	9386	2052	€ 6	€ 1	–€ 16	€ 20
Healthcare	Optimistic trial based	Trial costs	56316	12312	€ 39	€ 8	–€ 98	€ 119
Healthcare	Only F-ACT participants	Trial costs	48906	10692	€ 34	€ 7	–€ 85	€ 103

From the Third party payer perspective, no separate rate for guidance toward participation could be identified, and BPR and ACC were considered part of normal rates of care for this group of patients. Hence, from a health insurer perspective, no additional costs would occur if specialized mental health organizations started to use BPR more widely.

The BIA showed that from a societal perspective in scenario 1 (most optimistic regarding uptake of BPR), the expected budget impact in year 1 would be €101 million (95% IQR -€641to €870 million), and €22 million, (95%IQR -€140 million to €190 million) in year 2 and further. In scenario 2 (uptake as in the trial), the expected budget impact in year 1 would be €13 million (95% IQR -€81 million to €110 million) and €2.8 million (95% IQR -€18 million to €24 million) in year 2 and further. The two remaining scenarios had budget impact estimates in between these two extremes.

Results were mostly affected by differences in healthcare costs other than intervention costs of €685 (95% IQR -€7,794 to €9,535) and the difference in productivity costs of €665 (95% IQR -€670 to €1975). Differences in intervention costs were minor at around 2 euro per participant (95% IQR−130 to €129). All cost differences were highly uncertain, as reflected by the wide interquantile ranges, from cost savings to large additional costs.

## Discussion

Although BPR effectiveness regarding social participation has been confirmed in a number of RCT's when compared to standard care or an active control condition, dissemination and availability remain limited. This may be due to a lack of insight into the associated costs for MHC services providing this support, and into the benefits for society in terms of potential increases in quality of life and -if paid employment is obtained or kept- in productivity. Therefore, this study examined the cost effectiveness and budget impact of BPR in the area of social participation.

The main study investigating the effectiveness of BPR compared to ACC for individuals with SMIs in the area of social participation showed that both interventions effectively improved social participation after 12 months but no difference between the two interventions could be shown. Therefore, a priori expectations of BPR being cost-effective compared to ACC were low.

Results confirmed this, and BPR had a <50% probability of being cost-effective compared to ACC in the study population. Because the difference in QALYs was so small, the effects were not only statistically, but also clinically insignificant.

When costs collected over a 12 month follow-up period were divided into different types of expenditures, it became apparent that the highest total costs in both the intervention and control group were related to supported and sheltered housing, followed by inpatient care, outpatient care, and engaging in organized activities. The costs of supported and sheltered housing were considerably higher than those related to the other categories.

For the budget impact, the difference in intervention costs was small and non-significant, while differences in other healthcare costs and productivity costs were substantial, but highly uncertain. This resulted in an estimated budget impact that could range from cost savings to €190 million (see Table 3), depending on assumptions regarding uptake such as assuming participation of all eligible patients with SMIs aged 18–64, or only those who receive care from F-ACT (and variants in between). As there is no separate rate for support aimed at improving social participation, there would be no additional costs if BPR was implemented on a wider scale in mental health care institutions, from a health insurer perspective. This implies that any additional costs will have to be accommodated for by the mental healthcare organizations.

As this is the first study to investigate the cost-effectiveness of BPR in any goal area, it is difficult to compare the results to existing studies. Only studies targeted at paid employment have been conducted, and they have shown mixed results. For instance, Dixon et al. (37) compared IPS to enhanced vocational rehabilitation (EVR). The results were inconclusive as to whether IPS was more cost-effective than EVR. On the other hand, a study comparing IPS to standard vocational services (38) found that IPS had a high probability of being cost-effective. The general lack of cost effectiveness studies regarding psychiatric rehabilitation methods, and the mixed results of existing studies, clearly indicate that more research is needed in this area.

A number of strong points are associated with our study. First, an active control condition was used in which practitioners were explicitly instructed to offer support with rehabilitation goals. Many studies that investigate the effectiveness of protocolled rehabilitation methods use a control condition in which standard care but no active support is offered, which may overestimate the effect of protocolled rehabilitation methods. Second, studies on the cost-effectiveness of psychiatric rehabilitation programs often do not measure and analyze costs from a societal perspective, whereas we did. Including costs within as well as outside of the healthcare sector is important to providing appropriate incentives to take into account when making decisions on allocation or reimbursement of health-related resources (39, 40).Third, as far as we know, this is the first study to investigate the cost-effectiveness of a psychiatric rehabilitation approach focused on attaining a wide range of social participation goals as opposed to just competitive employment. Not all people with SMIs are willing to engage or capable of engaging in paid employment. It is therefore important to incorporate other forms of social activities in this type of study, such as unpaid employment. Fourth, the attrition rate was very low compared to other studies involving people with SMIs.

A limitation was the collection of cost data through interviews with service users, which could be considered less reliable than administratively recorded data or data obtained from public registries. However, production loss involving paid or unpaid employment is also difficult to calculate from registries. Furthermore, a study by Patel et al. (41) showed congruence between data obtained with self-reports and case records, and the TIC-P is a widely used self -report measure for the assessment of costs (42). A second limitation was the follow-up period of 12 months. A previous study on BPR effectiveness by a member of the current study group showed that rehabilitation goal attainment almost doubled in BPR from 12 to 24 months after enrolment (16). It should be noted that this study already found better results for BPR compared to ACC at 12 months. Perhaps more time was needed for psychiatric rehabilitation support, and perhaps BPR may need more time to show its full potential. RCTs in mental healthcare often have short follow-up periods for financial reasons. This may interfere with a reliable assessment of costs. Sharfstein and Clark (43) emphasize the importance of assessing costs over a sufficiently long period of time in order to include both short- and long-term outcomes. This may particularly apply to psychiatric rehabilitation methods as their effects may not be limited to the short-term but may become apparent with some delay. The National Health Care Institute even recommends a life-long time horizon for economic evaluations in healthcare (21). On the other hand, it could also be argued that generic health status is a relatively stable construct, which is not easily influenced by an increase in social participation. As data on this subject are lacking, it is difficult to predict what the effect of a longer follow-up period would be. A final limitation is that QALYs were derived from the SF-6D, which measures generic health and is not particularly tailored to individuals with SMIs (44, 45). In a study by Mulhern et al. (46), the SF-6D and the EuroQol-5D were found to be reliable in populations with common mental disorders, but possibly less sensitive to change for use among individuals with schizophrenia. On the other hand, a generic measure of quality of life allows broad comparison of results to other interventions and target groups.

Cost-effectiveness studies on psychiatric rehabilitation programs are scarce, while information on their effectiveness is needed in order to make decisions on further implementation. This is particularly important since MHC organizations continue to be faced with financial constraints. Future studies on psychiatric rehabilitation programs should therefore incorporate cost-effectiveness and budget impact analyses within a sufficiently long time horizon to fully capture the relevant costs and benefits. Future research should also focus on further exploring differences between subgroups to identify specific subgroups that may particularly benefit from BPR. Examples of such subgroup comparisons are unemployed or working in sheltered workshops compared to competitive employment or supported and sheltered housing compared to Independent living. Furthermore, the approach of “social return on investment” as the ratio of benefits (clients' work earnings) to total investment for each client, expressed as a percentage could be an interesting angle to explore.

The current study showed that BPR was not cost-effective compared to ACC. People in both conditions considerably increased their social participation, however at comparable costs. It would be unfair to discard the value of BPR based on a single cost-effectiveness study in a specific goal area. There were many factors that influenced the effectiveness of BPR compared to ACC such as the fact that ACC has become increasingly focused on rehabilitation in the past few years, and that most participants in our study sample wanted to obtain paid employment, which is the goal that proved the most difficult to obtain. These and other factors are extensively discussed in the main article on BPR effectiveness from the same study population (23). A process evaluation could have shed more light on the active components of successful psychiatric rehabilitation, and would be recommended for future studies.

As this was the first study to investigate the cost-effectiveness of BPR, and because we limited BPR to the area of social participation for which it was not specifically designed, more studies are needed before definite conclusions can be drawn on the cost-effectiveness of the method as a whole.

## Data Availability Statement

The datasets presented in this article are not readily available because restrictions apply to the availability of these data, which were used under license for this study. The data that support the findings of this study are available from the author WS, senior researcher, with the permission of Altrecht Institute for Mental Health Care. Requests to access the datasets should be directed to w.swildens@altrecht.nl

## Ethics Statement

The studies involving human participants were reviewed and approved by University Medical Center Groningen Ethical approval reference number 2013/70. The patients/participants provided their written informed consent to participate in this study.

## Author Contributions

SS, TF, WS, JvB, JvW, and TvA contributed to the study conception and design and commented on previous versions of the manuscript. SS, WS, JvB, TF, and TvA performed material preparation and data collection and analysis. SS wrote the first draft of the manuscript. All authors contributed to the article and approved the submitted version.

## Funding

This research was supported by a grant from the Netherlands Organization for Health Research and Development (ZonMw) (project number 837002006).

## Conflict of Interest

The authors declare that the research was conducted in the absence of any commercial or financial relationships that could be construed as a potential conflict of interest.

## Publisher's Note

All claims expressed in this article are solely those of the authors and do not necessarily represent those of their affiliated organizations, or those of the publisher, the editors and the reviewers. Any product that may be evaluated in this article, or claim that may be made by its manufacturer, is not guaranteed or endorsed by the publisher.

## Appendix

**Table A1:** Unit prices per type of costs in euros^1^.

**Cost category**	**Indexed unit price**
*Rehabilitation support*	
Psychiatric rehabilitation, including supervision^2^	
BPR ACC	143.66 (45 mins) 135.04 (45 mins)
Additional resources^3^	If used, than 736 (8 sessions at €92)
*Healthcare*	
MHC professionals:	
Psychiatrist Psychologist Case manager Social worker Group therapy (incl. recovery groups) Peer expert General practitioner Nurse practitioner Alternative healer Physiotherapist Visiting nurse Family support Spiritual counselor Other	96.61 (45 mins) 96.61 (45 mins) 106 (60 mins) 66.50 (60 mins) 61.38 (90 mins) 68.91 (60 mins) 33.76 (10 mins consultation) 17.39 (10 mins consultation) 57.63 (60 mins 19.53 (30 mins) 25.58 (60 mins) 23.96 (60 mins) 69.56 (60 mins) 12.79 (60 mins)
Hospitalizations:	
General hospital Academic hospital Psychiatric Forensic Revalidation Addiction	453.19 (per day) 656.77 (per day) 308.95 (per day) 350.51 (per day) 470.58 (per day) 308.95 (per day)
Supp./sheltered housing:	
Sheltered housing Case manager Care-taker Counselor in supported living^4^ Other	172.89 (a day, incl. personal counselor) 114.58 (per stay) 343.73 (per stay) 66.50 (per stay) 12.79 (per stay)
Outpatient visits:	
Outpatient clinic Day treatment General practice center First aid	93.09 (per visit) 282.35 (per visit) 90.77 (per visit) 264.96 (per visit)
Home care:	
Household Care Nursing	20.46 (60 mins) 51.15 (60 mins) 74.68 (60 mins)
Prescribed medication	Source: National Health Care Institute (27)
Unprescribed medication	Source: Health Care Institute (27)
*Costs outside healthcare*	
Organized activities	
Day activity center and drop in center	213.81 (per day)
Other	68.54 (per daily period)
Social support	€ 23.96 (60 mins)
Informal home care	12.79 (60 mins)
Out of pocket expenditures	N.A. (exact costs were reported in questionnaire)
*Indirect costs*	
Productivity loss unpaid work	14.13 (60 mins)
Productivity loss paid work	35.07 (60 mins)

1 *Reported costs are the unit prices that were used to calculate the total costs per category as reported in Table 2*;

2 *Same rate but supervision costs in BPR were somewhat higher*;

3 *Additional support such as external job coaching*;

4 *Rent is directly paid by patients, therefore the rate used is 0*.
